# Supplementary material for: Microglial expression of CD83 governs cellular activation and restrains neuroinflammation in experimental autoimmune encephalomyelitis
Source: Nat Commun. 2023 Aug 1;14:4601. doi: 10.1038/s41467-023-40370-2 (PMC10394088; doi:10.1038/s41467-023-40370-2)
Supplement: Supplementary file 1 — Supplementary Information [file 41467_2023_40370_MOESM1_ESM.pdf]

Sinner et al:
Microglial expression of CD83 governs cellular activation and restrains autoimmune neuroinflammation

Supplementary Data

Supplementary Figures & Legends 1-5

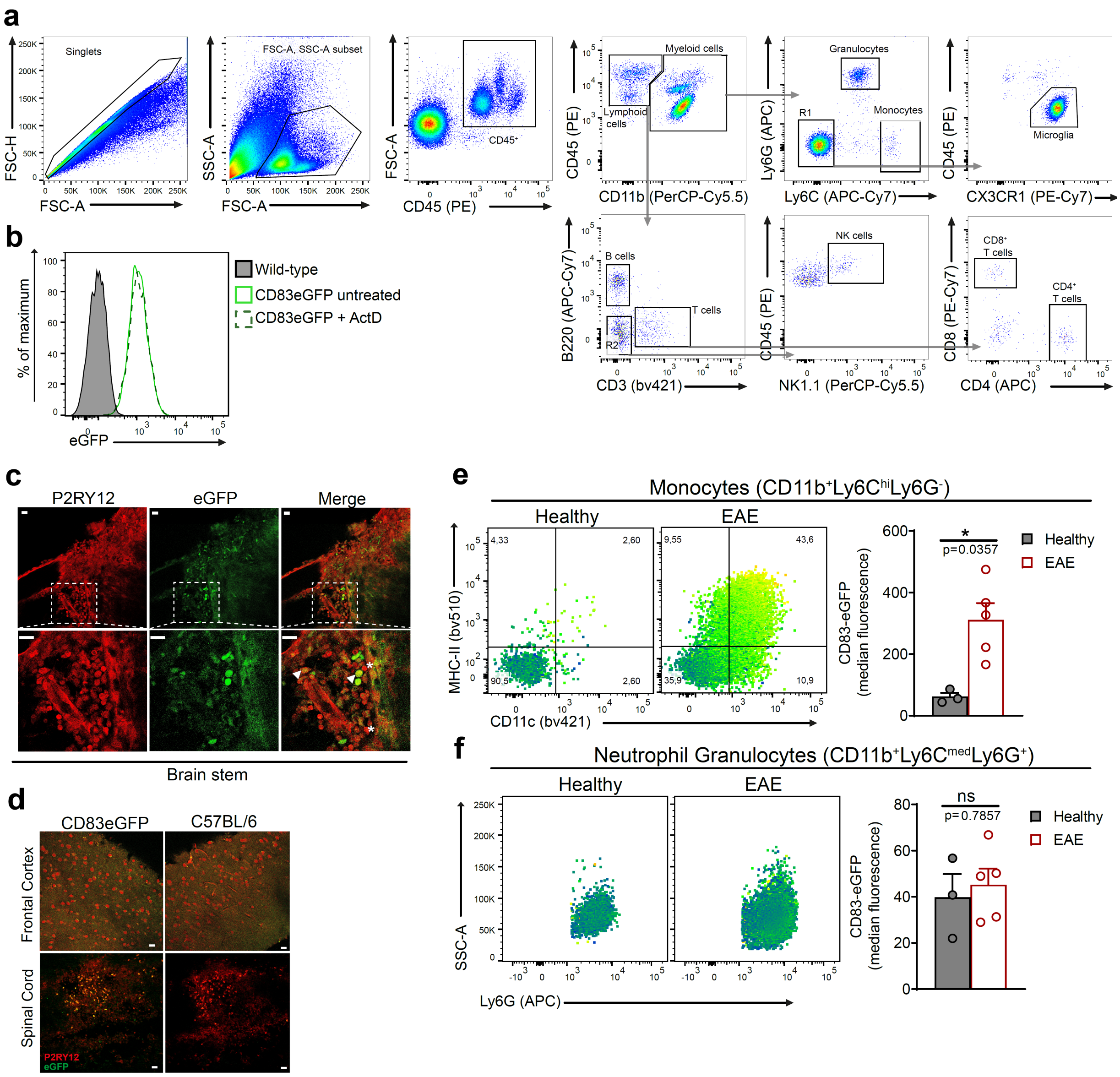

***Supplementary Figure 1:* Spatial and context-dependent expression of Cd83 in microglia**

a Representative gating strategy for immune cells in the CNS. **b** Histogram overlay of eGFP signal from microglia treated with actinomycin D. Microglia cells were isolated either from the CNS of wild-type or CD83-eGFP reporter mice. CD83e-GFP brains were divided into two different isolation media: for one hemisphere, 4 µM actinomycin D was added to all used solutions (i.e. RPMI, 1xHBSS, and 37 % Percoll); the other hemisphere was processed as stated in the methods section. Wild-type microglia served as negative fluorescence control. **c** Two-Photon-microscopy image of brainstems of CD83-eGFP mice isolated at the peak of EAE. White arrowheads indicate exemplified double labeling of cells with P2RY12, whereas representative GFP-negative microglia are labeled with white asterisks. Length of all scale bars: 20 µm **d** Two-Photon-microscopy images of frontal cortex and spinal cord from either CD83-eGFP or C57BL/6 wild-type mice. Only in the spinal cord, co-localization of P2RY12 and eGFP is visible (i.e. yellow cells). Length of all scale bars: 20 µm. **e** Distribution of eGFP-signal in monocytes isolated from the spinal cords either of healthy CD83-eGFP mice or at the peak of EAE. Representative dot plots of monocytes ($CD11b^{+}/CD45^{high}/Ly6C^{high}$), further gated on CD11c and MHC-II with color mapping to indicate CD83-eGFP fluorescence intensity, and quantitative analysis of eGFP-signal strength (n=3 for healthy and n=5 for EAE mice; pooled from two independent experiments). **f** Distribution of eGFP-signal in neutrophil granulocytes isolated from the spinal cords either of healthy CD83-eGFP mice or at the peak of EAE. Representative dot plots of granulocytes ($CD11b^{+}/CD45^{high}/Ly6C^{low}$), gated on SSC-A versus Ly6G with color mapping to indicate CD83-eGFP fluorescence intensity, and quantitative analysis of eGFP-signal strength (n=3 for healthy and n=5 for EAE mice; pooled from two independent experiments). Data are represented as mean ± SEM. Statistically significant differences were determined with two-tailed Mann-Whitney-U-test.

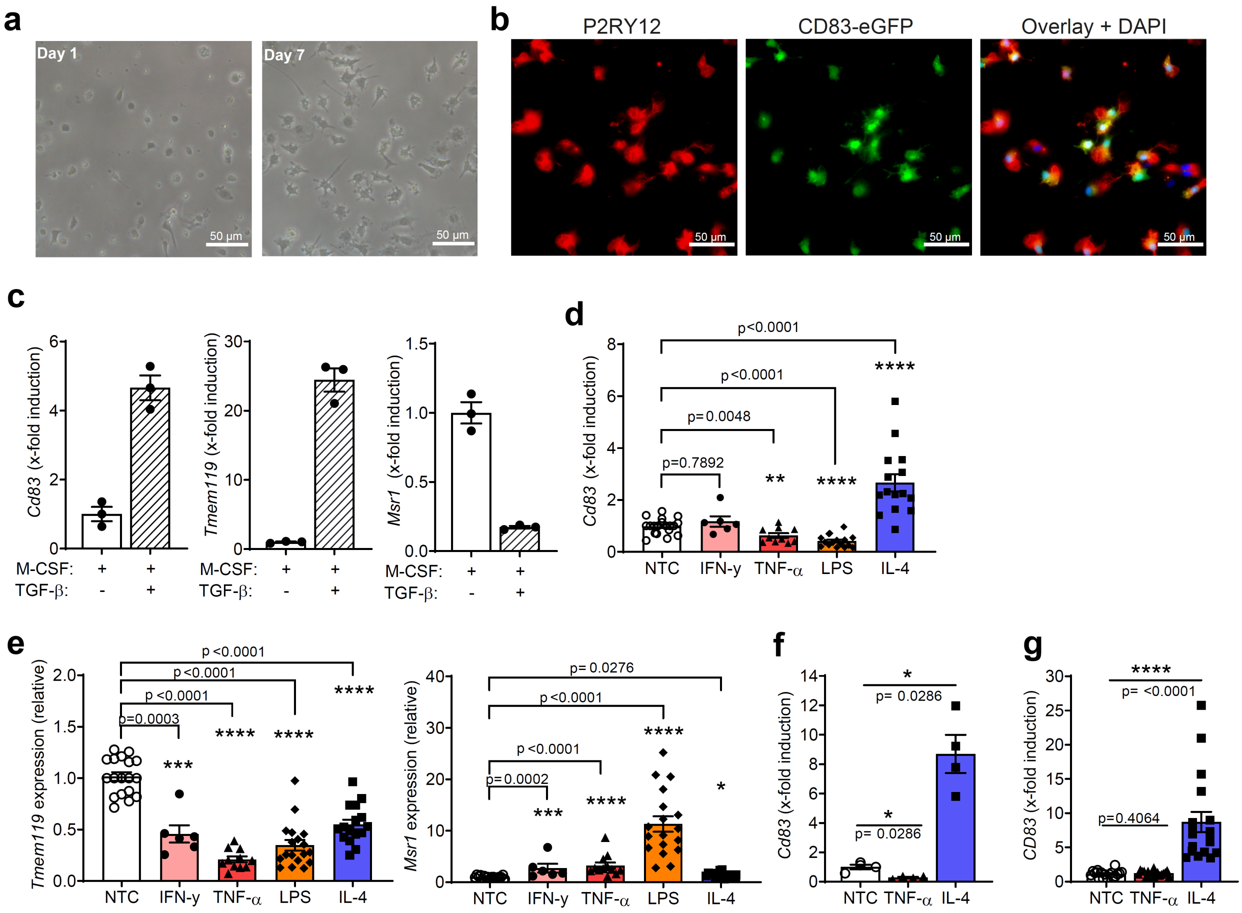

Supplementary Figure 2: Expression of CD83 in microglia depends on TGF-β signaling and is differentially regulated by pro- and anti-inflammatory stimuli

a Phase-contrast images of microglia cultures on day 1 and day 7 after isolation. **b I**mmunofluorescence images of microglial cultures originating from CD83-eGFP mice. Cells were stained on day 7 of culture using the microglia marker P2RY12. **c** Gene expression analysis of microglia cultivated in the presence or absence of 2 ng/ml TGF β for 7 days. Expression of *Cd83, Tmem119,* and *Msr1* was normalized to *Rpl4* as reference and is depicted as x-fold induction over M-CSF only cultures (n=3 individual mice). **d, e** Gene expression analyses in microglial cultures after different stimuli. Cells were treated on day 6 of culture with 300 U/ml IFN-γ (n=6), 500 U/ml TNF-α (n=12), 10 ng/ml LPS (n=18), or 40 ng/ml IL-4 (n=16) for 24 h. Expression of *Cd83* (**d**) or T*mem119* and *Msr1* (**e**) was normalized to *Rpl4* and is depicted as x-fold induction over non-treated cultures (NTC, n=20). Data are pooled from five independent experiments. **f** Change of *Cd83* expression in cultures of neonatal microglia after treatment with 500 U/ ml TNF-α or 40 ng/ml IL-4 for 24 h (n=4). **g** Expression analyses of *CD83* in human iPSC-derived microglia after treatment with either TNF-α or IL-4. Expression was normalized to *RPLP0* and is shown as x-fold induction over NTC (n=18, pooled from three different iPSC-lines). All data are represented as mean ± SEM. Two-tailed Mann-Whitney-U-test was used to analyze the data.

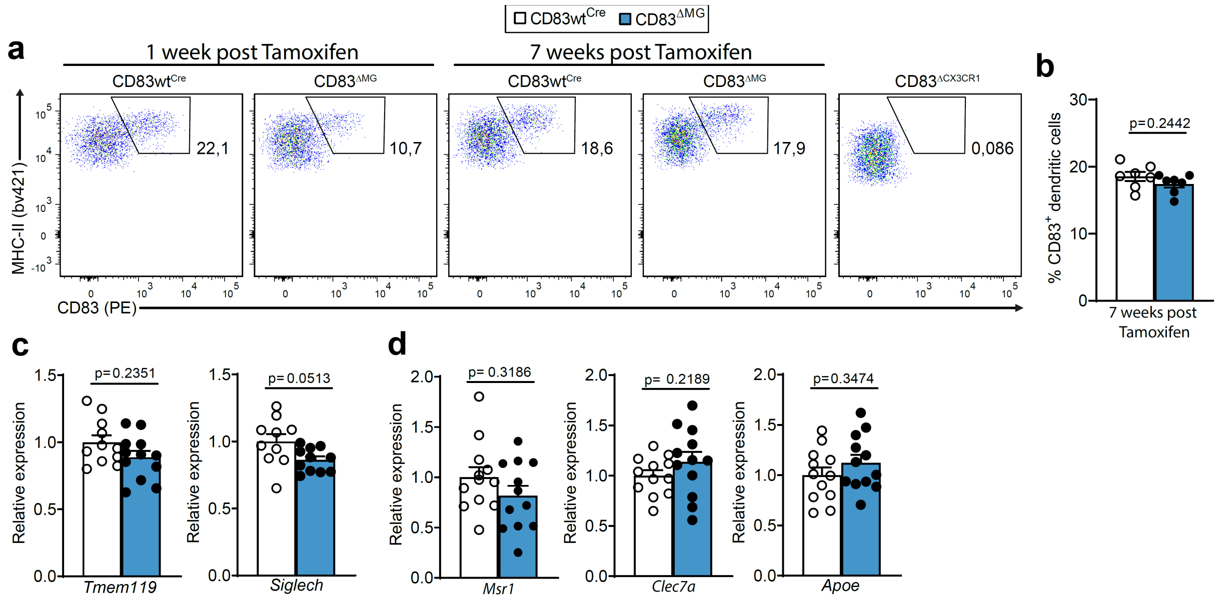

Supplementary Figure 3: Phenotypic analysis of CD83$^{\Delta MG}$ mice

a Flow cytometric analyses of splenic DCs from CD83$^{\Delta MG}$ and CD83wt^{Cre} mice on week or seven weeks after tamoxifen injection. Single cells were pre-gated on a B220^{-}, CD11c^{+}, and MHC-II^{high} subset, and CD83^{+} DCs were gated. Mice, in which CD83 is permanently deleted in all CX3CR1^{+} cells (CD83$^{\Delta CX3CR1}$) were used as positive control (rightmost panel). **b** Quantification of CD83^{+} splenic DCs 7 weeks after amoxifen treatment (n=7, pooled from two independent experiments). **c,d** Expression analyses of homeostatic (**c**) and disease-associated genes (**d**) in acutely isolated microglia (n=11 for CD83wt^{Cre} and n=12 for CD83$^{\Delta MG}$, pooled from three independent experiments). Data are represented as mean ± SEM. Statistically significant differences were determined with Mann-Whitney-U-test for all charts.

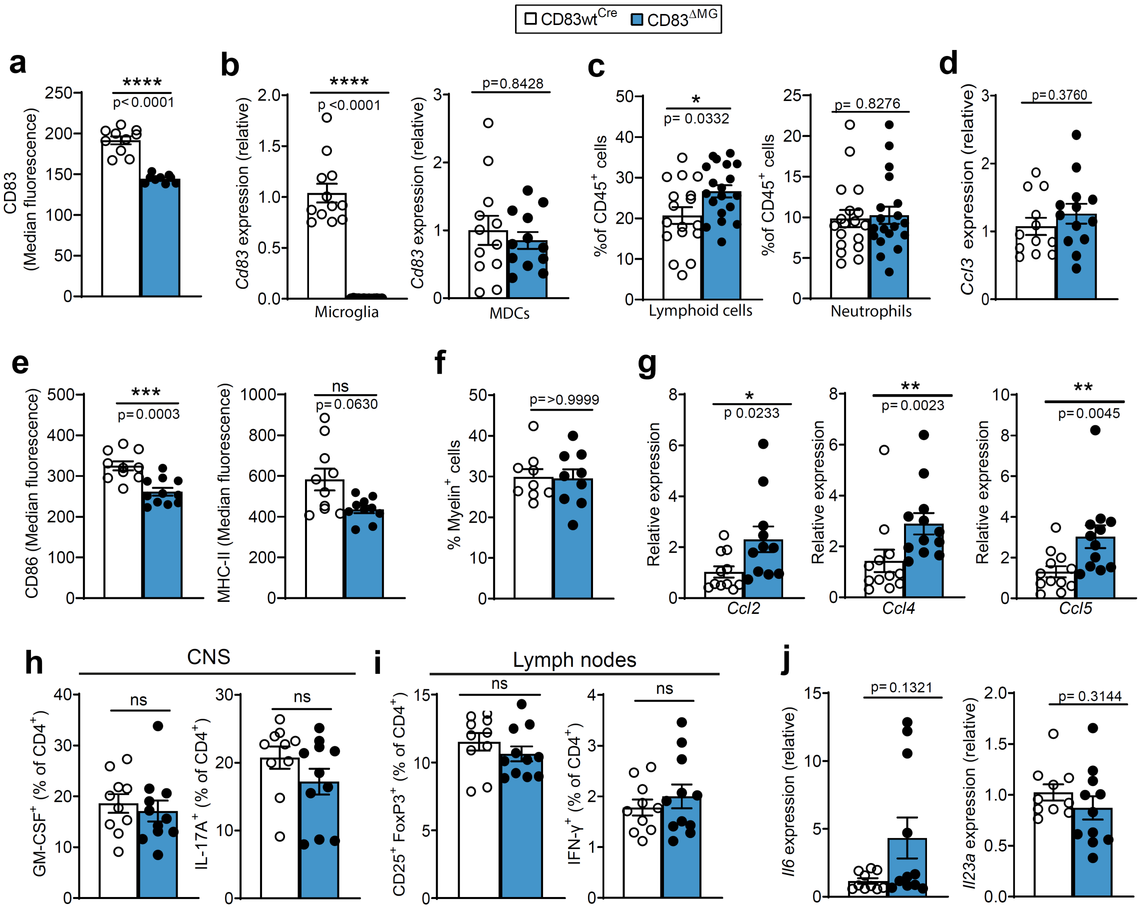

Supplementary Figure 4: Analyses of CD83$^{\Delta MG}$ mice during EAE

a Surface expression of CD83 in microglia from EAE mice at the peak of disease, i.e. day 18 (n=10 for CD83wt^{Cre} and n=9 for CD83$^{\Delta MG}$, pooled from 2 independent experiments). **b** Expression of *Cd83* on microglia and monocyte-derived cells (MDCs) at the peak of disease, i.e. day 18 p.i. (n=12, pooled from two independent experiments). **c** Perecentage of lymphoid cells (i.e. CD45^{+}/CD11b^{-}) and neutrophil granulocytes (CD11b^{+}/Ly6C^{int}/Ly6G^{+}) among al CD45^{+} cell within the CNS of EAE mice (n=18 for CD83wt^{Cre} and n=19 for CD83$^{\Delta MG}$, pooled from three independent experiments). **d** Expression levels of *Ccl3* on microglia at the peak of disease (n=12 for CD83wt^{Cre} and n=13 for CD83$^{\Delta MG}$, pooled from two independent experiments). **e** Flow cytometric analyses of CD86 and MHC-II on microglia cells at the peak of disease (n=10 for CD83wt^{Cre} and n=11 for CD83$^{\Delta MG}$, pooled from two independent experiments). **f** Percentage of cells positive for pHrodo-coupled myelin after 90 min incubation. Cells were pre-gated on CD11b^{+}/CD45^{low}/CX3CR1^{+} and cells incubated with myelin at 4 °C served as negative controls (n=9, pooled from three independent experiments). **g** Chemokine expression in MDCs at the peak of disease (n=12, pooled from two independent experiments). **h** Percentage of GM-CSF (left) and IL-17A (right) producing T cells. Single cell suspensions of EAE-CNS were re-stimulated with PMA/ionomycin for 5 h in the presence of golgi transport inhibitors and intracellularly stained for GM-CSF and IL-17A (n=10 for CD83wt^{Cre} and n=11 for CD83$^{\Delta MG}$, pooled from two independent experiments). **i** Proportions of CD25^{+}FoxP3^{+} Tregs and IFN-γ producing T cells in the inguinal draining lymph nodes of EAE animals at the peak of disease (n=10 for CD83wt^{Cre} and n=11 for CD83$^{\Delta MG}$, pooled from two independent experiments). **j** Gene expression of spinal cords at the peak of disease (n=10 for CD83wt^{Cre} and n=11 for CD83$^{\Delta MG}$, pooled from two independent experiments). Data are represented as mean ± SEM. Statistically significant differences were determined with Mann-Whitney-U-test for all charts.

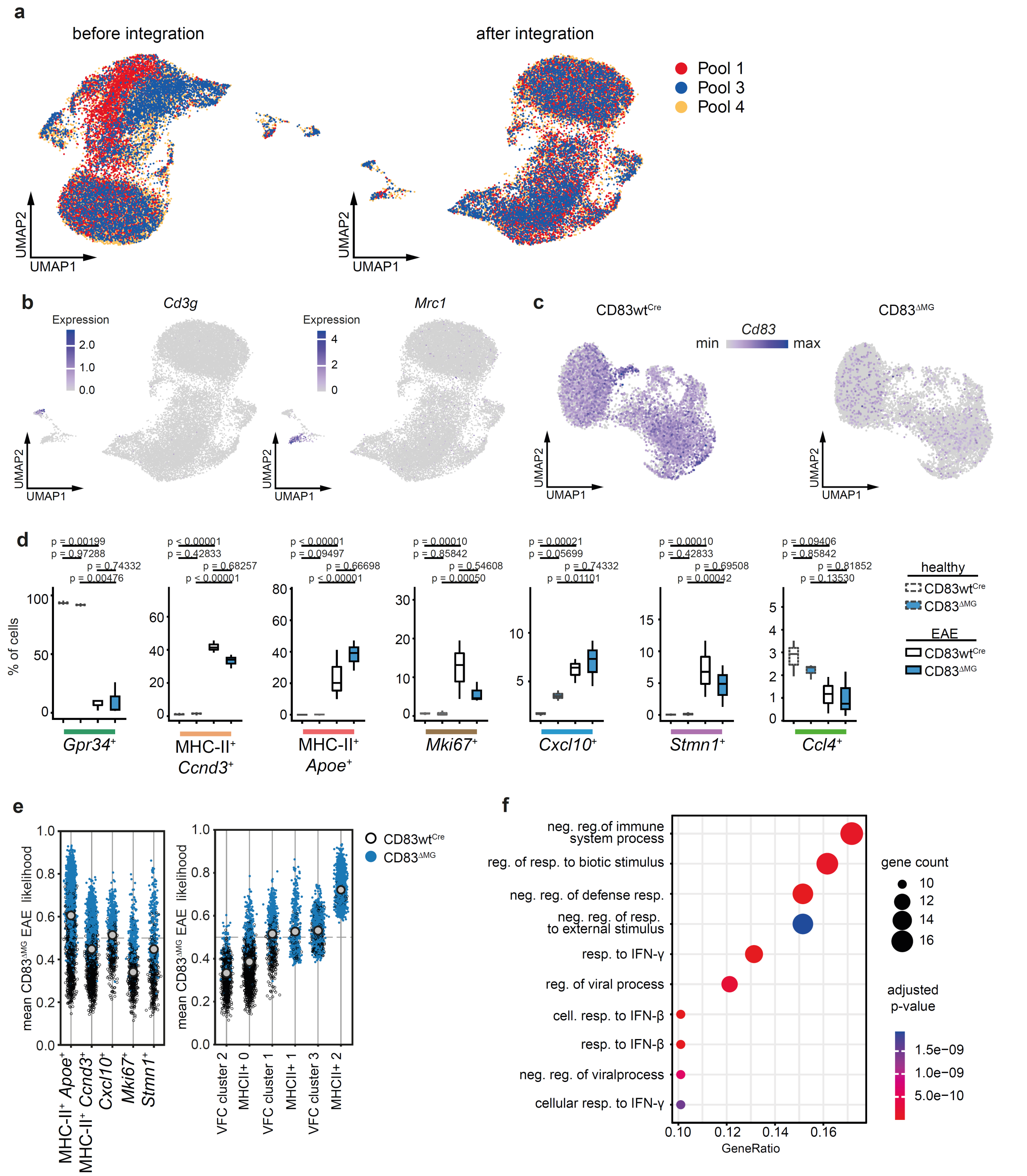

Supplementary Figure 5: Single-cell RNA-sequencing analyses of CD83$^{\Delta MG}$ mice during EAE (and homeostasis)

a U-map of scRNA-seq datasets before and after integration. Each pool represents a separate biological replicate library consisting of four individual animals, each from one of the following four conditions: healthy control CD83wt^{Cre}, healthy control CD83$^{\Delta MG}$, EAE CD83wt^{Cre}, EAE CD83$^{\Delta MG}$. Pool 2 was excluded after the initial QC. **b** U-maps of the integrated scRNA-seq datasets highlighting the expression of Cd3g and Mrc1 labelling T cells and macrophages, respectively. **c** U-maps of the integrated scRNA-seq datasets after removal of contaminating cells highlighting the expression of *Cd83* in CD83wt^{Cre} and CD83$^{\Delta MG}$ animals (independent of disease condition). **d** Differential abundance of microglia populations identified by Seurat's graph based clustering in healthy control CD83wt^{Cre}, healthy control CD83$^{\Delta MG}$, EAE CD83wt^{Cre}, EAE CD83$^{\Delta MG}$ animals. edgeR quasi-likelihood negative binomial generalized log-linear model F-test was used to test the differential abundance and corrected for multiple testing using the Benjamini-Hochberg method. Boxes and whiskers show median + quartiles + min-max; N = 3 per condition. **e** Relative likelihood for the microglia populations identified by Seurat's graph based clustering to be enriched in the EAE condition in CD83$^{\Delta MG}$ animals compared with CD83wt^{Cre} animals, and relative likelihood for each of the VFC clusters to be enriched in the EAE condition in CD83$^{\Delta MG}$ animals compared with CD83wt^{Cre} animals. **f** Heat-map of the top 10 Gene Ontology Biological Process pathways based on the top ten percent of genes correlating with *Cd83* expression in the EAE CD83wt^{Cre} condition. The correlation matrix was generated using the hdWGCNA workflow.
