# Supplementary material for: Microglial expression of CD83 governs cellular activation and restrains neuroinflammation in experimental autoimmune encephalomyelitis
Source: Nat Commun. 2023 Aug 1;14:4601. doi: 10.1038/s41467-023-40370-2 (PMC10394088; doi:10.1038/s41467-023-40370-2)
Supplement: Supplementary file 3 — Description of Additional Supplementary Files [file 41467_2023_40370_MOESM3_ESM.docx]

**Description of Additional Supplementary Files**

**Supplementary Movie 1: Steady-state environmental surveillance of cultured microglia:** Time-lapse photography of microglial cultures over 28 min. Photos were taken every minute and put together by the microscope adjacent software (Keyence Analyzer, frame-rate: 5 frames per second, fps). White arrow indicates on prominent cell, which is actively scanning its environment by dynamic movement of its protrusions.

**Supplementary Movie 2: Phagocytosis of pHrodo®-labeled myelin-debris:** Cultures of microglia were treated with pHrodo®-labeled myelin debris (20 µg/ml) and time-lapse picture were taken every 10 min for a total 5 hours. Movie was generated by microscope adjacent software with a frame-rate of 5 fps.
