# Supplementary material for: Microglial expression of CD83 governs cellular activation and restrains neuroinflammation in experimental autoimmune encephalomyelitis
Source: Nat Commun. 2023 Aug 1;14:4601. doi: 10.1038/s41467-023-40370-2 (PMC10394088; doi:10.1038/s41467-023-40370-2)
Supplement: Supplementary file 6 — Reporting Summary [file 41467_2023_40370_MOESM6_ESM.pdf]

nature portfolio

Corresponding author(s): Andreas B. Wild

Last updated by author(s): Jun 27, 2023

Reporting Summary

Nature Portfolio wishes to improve the reproducibility of the work that we publish. This form provides structure for consistency and transparency in reporting. For further information on Nature Portfolio policies, see our Editorial Policies and the Editorial Policy Checklist.

Statistics

For all statistical analyses, confirm that the following items are present in the figure legend, table legend, main text, or Methods section.

n/a	Confirmed	
☐	☒	The exact sample size (*n*) for each experimental group/condition, given as a discrete number and unit of measurement
☐	☒	A statement on whether measurements were taken from distinct samples or whether the same sample was measured repeatedly
☐	☒	The statistical test(s) used AND whether they are one- or two-sided<br>*Only common tests should be described solely by name; describe more complex techniques in the Methods section.*
☒	☐	A description of all covariates tested
☐	☒	A description of any assumptions or corrections, such as tests of normality and adjustment for multiple comparisons
☐	☒	A full description of the statistical parameters including central tendency (e.g. means) or other basic estimates (e.g. regression coefficient) AND variation (e.g. standard deviation) or associated estimates of uncertainty (e.g. confidence intervals)
☐	☒	For null hypothesis testing, the test statistic (e.g. *F*, *t*, *r*) with confidence intervals, effect sizes, degrees of freedom and *P* value noted<br>*Give P values as exact values whenever suitable.*
☒	☐	For Bayesian analysis, information on the choice of priors and Markov chain Monte Carlo settings
☒	☐	For hierarchical and complex designs, identification of the appropriate level for tests and full reporting of outcomes
☒	☐	Estimates of effect sizes (e.g. Cohen's *d*, Pearson's *r*), indicating how they were calculated

Our web collection on statistics for biologists contains articles on many of the points above.

Software and code

Policy information about availability of computer code

Data collection	BD FACSDiva Software v8.0.1
Data analysis	FlowJo V10.7, BioRad CFX Maestro V 2.3, GraphPad Prism 9.3.1, Seurat 4.1.1, R 4.2.1, edgeR 3.38.1, Python 3.8.8, Clusterprofiler 4.4.4, hdWGCNA 0.2.02, 10x Genomics Cell Ranger pipeline 6.0.0, SCTransform (v2),

For manuscripts utilizing custom algorithms or software that are central to the research but not yet described in published literature, software must be made available to editors and reviewers. We strongly encourage code deposition in a community repository (e.g. GitHub). See the Nature Portfolio guidelines for submitting code & software for further information.

Data

Policy information about availability of data

All manuscripts must include a data availability statement. This statement should provide the following information, where applicable:

- Accession codes, unique identifiers, or web links for publicly available datasets
- A description of any restrictions on data availability
- For clinical datasets or third party data, please ensure that the statement adheres to our policy

Data of scRNASeq are available under accession code GSE230753.
Source data of all experiments are provided with this paper.

Research involving human participants, their data, or biological material

Policy information about studies with human participants or human data. See also policy information about sex, gender (identity/presentation), and sexual orientation and race, ethnicity and racism.

Reporting on sex and gender	Sex and/or gender were not considered for selecting the representative tissue section shown in Fig 1e
Reporting on race, ethnicity, or other socially relevant groupings	Race or ethnicity were not considered in this study
Population characteristics	This study used access cortical tissue outside the tumor area, resected in order to gain access to the pathology, obtained from a patient undergoing tumor surgery. The patient was female and 60 years old.
Recruitment	N/A
Ethics oversight	Use of spare access cortical tissue was in accord with the terms of the informed consents. Fully informed consent was obtained for the tissue donation under ethical approval by the Ethics Committee of the University of Tübingen (Reference# 147/2021BO2).

Note that full information on the approval of the study protocol must also be provided in the manuscript.

Field-specific reporting

Please select the one below that is the best fit for your research. If you are not sure, read the appropriate sections before making your selection.

☒ Life sciences ☐ Behavioural & social sciences ☐ Ecological, evolutionary & environmental sciences

For a reference copy of the document with all sections, see nature.com/documents/nr-reporting-summary-flat.pdf

Life sciences study design

All studies must disclose on these points even when the disclosure is negative.

Sample size	No statistical methods were used to pre-determine sample sizes. Sample size used in in vivo experiments was informed by our previous work in autoimmune responses in CNS Wherever statistical tests were applied, we used at least n=4 to ensure non-parametric testing (except for Fig. 2f and Fig. 3c, where no statistical tests were applied due to n=3).
Data exclusions	In some datasets significant outliers were detected using Grubbs test (alpha=0.05). See Source data for further information.
Replication	All experiments were successfully repeated at least twice.
Randomization	There was no allocation of test subjects in this study and thus, randomization was not applicable
Blinding	The genotype of the mice was blinded to the investigator performing scoring of the EAE mice. The genotype of the mice was unkown to the technicians performing cell culture, stimulations, RNA-isolation, and qPCR. For gene expression analyses in human microglia cultures, blinding was not necessary because no subjective methods (such as scorings or manual counting) were applied.

Reporting for specific materials, systems and methods

We require information from authors about some types of materials, experimental systems and methods used in many studies. Here, indicate whether each material, system or method listed is relevant to your study. If you are not sure if a list item applies to your research, read the appropriate section before selecting a response.

Materials & experimental systems

n/a	Involved in the study	
☐	☒	Antibodies
☐	☒	Eukaryotic cell lines
☒	☐	Palaeontology and archaeology
☐	☒	Animals and other organisms
☒	☐	Clinical data
☒	☐	Dual use research of concern
☒	☐	Plants

Methods

n/a	Involved in the study	
☒	☐	ChIP-seq
☐	☒	Flow cytometry
☒	☐	MRI-based neuroimaging

Antibodies

Antibodies used

Mouse antibodies
B220 (RA3-6B2, rat IgG, Biolegend #103224; 1:200 dilution)
CD3 (17A2, rat IgG, Biolegend #100228; 1:200 dilution)
CD4 (RM4-5, rat IgG, Biolegend #100568; 1:200 dilution)
CD8 (53-6.7, rat IgG, Biolegend #100722; 1:100 dilution)
CD11b (M1/70, rat IgG, Biolegend #101228; 1:100 dilution),
CD11c (N418, Armenian hamster IgG, Biolegend #117343; 1:100 dilution),
CD25 (PC61, rat IgG, Biolegend #102006; 1:200 dilution),
CD45 (I3/2.3, rat IgG, Biolegend #147710; 1:100 dilution),
CD83 (Michel19, rat IgG, Biolegend #121508; 1:50 dilution),
CD86 (GL-1, rat IgG, Biolegend #105030; 1:200 dilution),
CX3CR1 (SA011F11, mouse IgG, Biolegend #149016; 1:200 dilution),
FoxP3 (FJK-16s, rat IgG, Thermo Fisher Scientific #50-5773-80; 1:100 dilution),
IFN-γ (XMG1.2, rat IgG, Biolegend #505826; 1:200 dilution),
IL-17A (TC11-18H10.1, rat IgG, Biolegend #506912; 1:300 dilution),
I-A/I-E (M5/114.15.2, rat IgG, Biolegend #107635; 1:200 dilution),
Ly6C (HK1.4, rat IgG, Biolegend #128026; 1:400 dilution),
Ly6G (1A8, rat IgG, Biolegend #127614; 1:100 dilution),
P2RY12 (S16007D, rat IgG, Biolegend #848005; 1:100 dilution),

Human antibodies
Iba1 (goat polyclonal, Novus Biologicals #NB100-1028, 1:250 dilution),
CD83 (HB15a, mouse IgG, Beckman Coulter #IMBULK1C, 1:250 dilution)

Further antibodies:
goat-anti-rat-AlexaFluor594 antibody (clone Poly4054, Biolegend #405422, 1:250 dilution)
donkey-anti-mouse-AlexaFluor488 (Jackson ImmunoResearch #715-545-150, 1:250 dilution)
donkey-anti-goat-AlexaFluor568 (Invitrogen, #A11057, 1:250 dilution)

Validation

The validation of all primary commercial antibodies for the species and application was warranted by the vendors. Validation statements can be found on the manufacturers' website:

B220: https://www.biolegend.com/en-us/products/fitc-anti-mouse-human-cd45r-b220-antibody-445
CD3: https://www.biolegend.com/en-us/products/brilliant-violet-421-anti-mouse-cd3-antibody-7326
CD4: https://www.biolegend.com/en-us/products/apc-cyanine7-anti-mouse-cd4-antibody-1937
CD8: https://www.biolegend.com/en-us/products/percp-anti-mouse-cd8a-antibody-4256
CD11b: https://www.biolegend.com/en-us/products/percp-anti-mouse-human-cd11b-antibody-4315
CD11c: https://www.biolegend.com/en-us/products/brilliant-violet-421-anti-mouse-cd11c-antibody-7149
CD25: https://www.biolegend.com/en-us/products/fitc-anti-mouse-cd25-antibody-422
CD45: https://www.biolegend.com/en-us/products/fitc-anti-mouse-cd45-antibody-9796
CD86: https://www.biolegend.com/en-us/products/apc-cyanine7-anti-mouse-cd86-antibody-6554
CX3CR1: https://www.biolegend.com/en-us/products/pe-cyanine7-anti-mouse-cx3cr1-antibody-11909
FoxP3: https://www.thermofisher.com/antibody/product/FOXP3-Antibody-clone-FJK-16s-Monoclonal/606-5773-82
IFN-γ: https://www.biolegend.com/en-us/products/pe-cyanine7-anti-mouse-ifn-gamma-antibody-5865
IL-17A: https://www.biolegend.com/en-us/products/alexa-fluor-647-anti-mouse-il-17a-antibody-3536
I-A/I-E: https://www.biolegend.com/en-us/products/brilliant-violet-510-anti-mouse-i-a-i-e-antibody-7997
Ly6C: https://www.biolegend.com/en-us/products/apc-cyanine7-anti-mouse-ly-6c-antibody-6758
Ly6G: https://www.biolegend.com/en-us/products/apc-anti-mouse-ly-6g-antibody-6115
P2RY12: https://www.biolegend.com/en-us/products/apc-anti-p2ry12-antibody-15402
Iba1:https://www.novusbio.com/products/aif-1-iba1-antibody_nb100-1028#ReviewsSection

murine CD83 (Michel19): was validated in the indicated dilution on CD83-deficient dendritic cells in our laboratory
human CD83 (HB15a): was validated in the indicated dilution on human moDCs (immature versus mature cells)

Eukaryotic cell lines

Policy information about cell lines and Sex and Gender in Research

Cell line source(s)

KOLF2.1: The Jackson Laboratory; Sex: male
BIONi010-C: European Bank for Induced Pluripotent Stem Cells; sex: male
BIONi037-A: European Bank for Induced Pluripotent Stem Cells; sex: female

Authentication

Authentication of all cell lines has been previously described (DOI:10.1016/j.stem.2022.11.004; https://ebisc.org/BIONi010-C; https://ebisc.org/BIONi037-A) and the identity of cell lines was routinely confirmed by Sanger sequencing

Mycoplasma contamination

All cell lines were routinely checked for mycoplasma using Venor GeM kit. All cell lines tested negative for mycoplasma contamination.

Commonly misidentified lines
(See ICLAC register)

N/A

Animals and other research organisms

Policy information about studies involving animals; ARRIVE guidelines recommended for reporting animal research, and Sex and Gender in Research

Laboratory animals	We used B6.129P2(C)-Cx3cr1tm2.1(cre/ERT2)Jung/J mice (purchased from Jackson) which were bred together with Cd83tm1.1Lnjt, which were established in our laboratory. In addition, STOCK Tg(Cx3cr1-cre)MW126Gsat/Mmucd mice bred to Cd83tm1.1Lnjt were used. The age of the animals ranged from 12 to 16 weeks to assure repopulation of CD83-expressing peripheral immune cells.
Wild animals	The study did not involve wild animals.
Reporting on sex	We used an equal number of both sexes in the experiments, also with respect to adopting the 3R-principle (reduction of animals).
Field-collected samples	The study did not involve field-collected samples.
Ethics oversight	The present study was performed in accordance with the European Community Standards on the Care and Use of Laboratory Animals and were approved by the Administration of Lower Franconia (Reference number 55.2.2-2532-2-1193).

Note that full information on the approval of the study protocol must also be provided in the manuscript.

Flow Cytometry

Plots

Confirm that:

☒ The axis labels state the marker and fluorochrome used (e.g. CD4-FITC).

☒ The axis scales are clearly visible. Include numbers along axes only for bottom left plot of group (a 'group' is an analysis of identical markers).

☒ All plots are contour plots with outliers or pseudocolor plots.

☒ A numerical value for number of cells or percentage (with statistics) is provided.

Methodology

Sample preparation	Microglia and other immune cells within the CNS were isolated by a one-step density centrifugation protocol to remove myelin debris. Cells were stained in PBS + 1 mM EDTA and the respective antibody mixes.
Instrument	BD FACS Canto II
Software	The FACS Canto was running the BD FACS-Diva Software.<br>Data were evaulated using FlowJo V10
Cell population abundance	In healthy mice, we acquired 20,000 cells in the CD45+ gate for further analysis. In EAE mice, 50,000 cells were acquired in the CD45+ gate.
Gating strategy	For all experiments, we first excluded doublets via FSC-A vs FSC-H. Then, we gated on living cells via FSC-A vs SSC-A, followed by gating on CD45 positive cells to select immune cells. Further populations were gated:<br>-Microglia: CD45low, CD11b positive, CX3CR1 positive<br>-Monocytes: CD45positive, CD11b positive, Ly6C positive, Ly6G negative<br>-Neutrophil granulocytes:CD45positive, CD11b positive, Ly6C positive, Ly6G positive<br>-Lymphocytes: CD45positive, CD11b negative:<br>-B cells: B220 positive<br>-CD4+ T cells: CD3 positive, CD4 positive<br>-CD8+ T cells: CD3 positive, CD8 positive

☒ Tick this box to confirm that a figure exemplifying the gating strategy is provided in the Supplementary Information.
